# DNA methylation, but not microRNA expression, is affected by in vitro THC exposure in bovine granulosa cells

**DOI:** 10.1186/s40360-024-00763-5

**Published:** 2024-07-15

**Authors:** Sabrina Floccari, Reem Sabry, Laurie Choux, Michael S. Neal, Jibran Y. Khokhar, Laura A. Favetta

**Affiliations:** 1 Reproductive Health and Biotechnology Lab, Department of Biomedical Sciences, Ontario Veterinary College, University of Guelph, Guelph, ON Canada; 2 ONE Fertility, Burlington, ON Canada; 3 Department of Obstetrics and Gynecology, McMaster University, Hamilton, ON Canada; 4 Department of Anatomy and Cell Biology, Western University, London, ON Canada

**Keywords:** Cannabis, THC, Granulosa cells, Fertility, DNA methylation, MicroRNAs

## Abstract

**Background:**

A global increase in cannabis use has led to questions about its effects on fertility. The rise in consumption amongst women of reproductive age is a growing concern, as this group is vulnerable in terms of reproductive health. Ample evidence suggests that the psychoactive component of cannabis, Δ^9^-Tetrahydrocannabinol (THC), interacts with the endocannabinoid system (ECS), that helps regulate mammalian reproduction. This study aimed to research the epigenetic effects of THC in bovine granulosa cells (GCs) by (1) investigating global DNA methylation via measuring 5-mC and 5-hmC levels; (2) measuring key methylation regulators, including the methylating enzymes *DNMT1, DNMT3a, DNMT3b* and the demethylases *TDG* and *TET1/2/3*; and (3) assessing fertility-associated miRNAs key in developmental competency, including miR-21, -155, -33b, -324 and -346.

**Methods:**

Bovine GCs were used as a translational model for reproductive toxicity in humans. To determine THC effects, GCs were isolated from Cumulus-Oocyte-Complexes (COCs) from bovine ovaries, cultured in vitro for 7 days, or until confluent, and cryopreserved at passage 1 (P1). For experimentation, cells were thawed, cultured until passage 2 (P2), serum restricted for 24-h and treated for 24-h in one of five groups: control, vehicle (1:1:18 ethanol: tween: saline) and three clinically relevant THC doses (0.032, 0.32 and 3.2 μM). Global methylation was assessed by measuring 5-mC and 5-hmC levels with flow cytometry. To assess mRNA and protein expression of methylation regulators and miRNA profiles, qPCR and Western Blotting were utilized. Shapiro-Wilk test was used to determine normality within datasets. One-way ANOVA was applied to determine statistical significance using GraphPad Prism 6.0.0.

**Results:**

Results indicate a significant decrease (*p* = 0.0435) in 5-mC levels following low THC exposure, while no changes were observed in 5-hmC levels. A significant increase in *DNMT1* following high THC exposure at the RNA level (*p* < 0.05) and a significant increase following low THC exposure at the protein level (*p* = 0.0048) were also observed. No significant differences were observed in *DNMT3a/3b, TDG, TET1/2/3* mRNAs or in any of the miRNAs analyzed.

**Conclusions:**

This research suggests that THC mainly affects DNA methylation, but not miRNA profiles, ultimately altering gene expression and likely impairing oocyte competence, maturation, and fertilization potential.

**Supplementary Information:**

The online version contains supplementary material available at 10.1186/s40360-024-00763-5.

## Background

Infertility affects approximately 1 in 6 adults, or about 17.5% of the population worldwide [1] and it is clinically defined as the inability to achieve pregnancy after 12 months of unprotected sexual intercourse. Approximately 37% of infertility cases have been linked to female, 29% to male, and 18% to both reproductive systems [2], while over 25% of cases have no known cause [3, 4]. Causes for female infertility include ovulatory, tubal, uterine or hormone disorders [4, 5]. Factors contributing to ovulatory disorders include polycystic ovary syndrome (PCOS), endocrine disorders, maternal age, lifestyle and environmental factors [6, 7]. Couples’ experiencing infertility often resort to Assisted Reproductive Technology (ART) leading to 3.2 million ART cycles performed annually at a 10% annual growth rate, and more than 9 million babies born using this technology [1, 8]. Still, improvements are needed, as the success rate for each cycle is only approximately 25% [9]. With the decline in global fertility, and the overall low ART success rates, there is a growing interest in identifying potential environmental and social factors contributing to fertility disorders [2].

The effects of recreational drugs and alcohol use on health and reproduction has been linked to several human disorders [2, 10]. Cannabis is now one of the most accepted and widely used drugs in the Western world [11–15]. The perceived risk of cannabis has declined with its increased acceptance [16], leading to aclimb in cannabis consumption. Furthermore, a study in Colorado found 70% of dispensaries recommended cannabis use to pregnant women for treating nausea, while 36% stated using cannabis was safe during pregnancy [17]. With a large support from the public and media attention, it is not surprising that The National Institute on Drug Abuse found that cannabis use among young adults (ages 19–30) has risen by 13.2% from 2011 to 2021 [18]. In Canada, the percentage of young adults reporting cannabis use in 2021 was 37% and 49% between the ages of 16–19 and 20–24, respectively [16]. The majority of Canadian users (>90%) also reported cannabis having no effect or being beneficial in their social lives, personal relationships, mental and physical health, home life, school and work performance [16] showing that cannabis users mainly have positive attitudes when evaluating the effects of cannabis on their health and wellness. Likewise, Canadian youth have documented multiple reasons for using cannabis, including social anxiety, identity formation, social acceptability, perceived acceptability, and the lower perceived risk when compared to other substances [19]. In addition to the higher frequency of cannabis consumption, Δ^9^-Tetrahydrocannabinol (THC) potency in cannabis has also drastically increased from 4% in 1995 [11] to 20.5% in 2022 [20, 21]. Likewise, the ratio of THC to cannabidiol (CBD) has drastically risen over the last 20 years [22, 23]. The rise in THC potency in recreational cannabis products can be attributed to increased customer demand of products containing high THC. It can also be attributed to changes in cultivation practices and technological advances, such as indoor hydroponic cultivation, cross breeding, genetic manipulations and in general, improved access to potent seeds [24].

Recreational cannabis is derived from the plant *cannabis sativa*, consisting of rich metabolites including phytocannabinoids THC and CBD. Different intake methods and the type of cannabis consumed causes various psychological effects [25]. THC is the main psychoactive component in cannabis and elicits its effects via the endocannabinoid system (ECS), an important modulatory system responsible for maintain homeostasis, regulating energy balance, lipid metabolism, cell growth, immune functions, and reproductive physiology [15, 26–28]. The ECS comprises endocannabinoids (eCBs), such as anandamide (AEA) and 2-arachidonyl glycerol (2-AG), cannabinoid receptors CB1 and CB2, and the enzymes involved in their synthesis, breakdown, and transportation. The CB1 and CB2 receptors are grouped under the transmembrane-spanning G-protein coupled family of receptors [25]. Their activation results in downstream cellular physiological changes [25, 27, 29–33], such as lowered cAMP and AC levels, initiation of mitogen-activated protein (MAP) kinases, and inhibition of calcium channels [27, 32, 34–36].

THC can directly modulate the ECS, which has been detected throughout the female reproductive system including the placenta, uterus, endometrium, ovary, embryo, oocyte, follicular fluid and granulosa cells (GCs) [15, 37–40]. The ECS controls various aspects of reproduction, including the release of gonadotropins, steroid hormone synthesis, the production and release of male and female gametes, and pregnancy [15, 41]. The primary receptor involved in the cellular response to THC, CB1, has been detected in the ovary, oviduct, uterus and placenta [41]. The regulation of eCBs is important to reproductive success, thus, enhanced ECS signalling by exogenous cannabinoids may impair fertility [27]. This is especially concerning with the rise in young adults and expecting mothers using cannabis [42]. It is therefore critical to further study the potential effects of cannabis on oocyte competency, maturation, and pre-implantation embryonic development [43, 44].

More recently, experts have questioned how THC exposure to gametes could impair fertility. Although some research has identified no conclusive link between cannabis use and female reproductive health [45, 46], some studies have found effects to placental formation [47–49], altered LH and FHS levels [50, 51], fewer oocytes and with lower quality, lower *in vitro* fertilization (IVF) success rates [52], reduced fecundability [53] and disruption to early embryonic development and maturation [54]. A study by Ryan et al. [51] found increased menstrual cycle length and basal FSH concentrations in response to increased THC concentrations in the rhesus macaques, suggesting ovulatory disruption. Similarly, THC-exposed GCs had increased proliferation, decreased apoptosis and increased VEGF and PGE2 secretion, which have been associated with ovarian dysfunction [55]. Furthermore, THC can affect placental development [49]. 24-h THC exposure increased NAPE-specific phospholipase D (NAPE-PLD) and decreased Fatty acid amine hydrolase (FAAH) levels in human placenta. Interestingly, 72 h treatment showed an opposite trend of decreased NAPE-PLD, increased FAAH levels, and increased AEA levels [56]. Chang et al. [47] showed that THC inhibited trophoblast cell migration and invasion by activating the STAT3 signaling pathway, ultimately affecting placental development. They also found, using cannabinoid receptor inhibitors, that THC dysregulated trophoblast function partly through CB1 and CB2 receptors [47].

Recent studies have focused on prenatal cannabis exposure and heritable alterations in the genome [11, 12, 57]. Epigenetic mechanisms plays a role in regulating gene expression and can be a source of heritable changes from cannabis exposure [11]. Additionally, cross-generational impacts of environmental insults are assumed to be mediated through epigenetic mechanisms [13, 33]. DNA methylation is an epigenetic mechanism that is highly involved in regulating gene expression and is vulnerable to environmental stressors [13, 58]. DNA methyltransferases (DNMTs), such as *DNMT1, DNMT3a* and *DNMT3b*, mediate DNA methylation by maintaining methyl marks and establishing de novo DNA methylation patterns. Demethylation is regulated by a second group of enzymes: Ten-eleven translocation *(TETs) (TET1/2/3)* and Thymine DNA glycosylase *(TDG).* Several studies have reported cannabis causing epigenetic dysregulation [33, 59], with 6640 differentially methylated CpG sites in human sperm between cannabis users versus non-users [11]. Enriched CpG sites were associated with Hippo signaling pathways and pathways in cancer, which they further replicated in THC exposed rodents, indicating THC may be causing these epigenetic modifications [11]. A study by Fuchs Weizman et al. [57] found a decrease in *DNMT3b* (de novo methylator) both in vivo and in vitro and decreased global methylation patterns in vitro, in GCs following THC exposure. miRNAs are other epigenetic factors important in development and are affected by cannabis [60, 61]. Martίnez-Peña et al. [55] found prenatal THC exposure resulted in differentially expressed miRNAs, including miR-122-5p in the rat ovary. Moreover, a transcriptomic analysis in our lab showed altered miRNA expression in THC exposed sperm [62]. The epigenome thus offers insight into the effects of environmental stressors on changes at the cellular level, based on altered signalling pathways and gene expression.

As the goal for our research is to ultimately analyze epigenetic patterns in the oocyte and embryo, which cannot be performed in human samples, we used bovine cells as a translational model. The bovine species is an excellent translational model for human reproduction as bovine and humans share similarities in ovarian function, oocyte characteristics, metabolic requirements, genome activation and embryo development [3], and humans and cows are both single ovulators. Additionally, Rodriguez-Osorio et al. [63] showed that DNMTs have a higher degree of conservation in their protein sequence between bovine and humans compared to other species.

In this study, fertility-associated miRNAs, including miR-21, miR-155, miR-346, miR-33b and miR-324 were assessed. miR-21 is highly expressed in murine GCs [64], ovine follicles [65], and is upregulated during human ovulation [66]. miR-21 regulates transcripts involved in cell cycle and apoptosis [66], therefore promoting follicular cell survival during ovulation [67], and its suppression leading to apoptosis in GCs [64]. miR-155 is another key miRNA as its dysregulation leads to ovulatory pathology such as PCOS [67, 68]. By regulating *PDCD4*, miR-155 activates the PI3K/AKT and JNK pathways, promoting cell proliferation, migration and invasion [67]. This is particularly interesting as PI3K/AKT pathways are also modulated by the ECS. Another miRNA linked to developmental competency is miR-33b which was found to be upregulated in GCs of PCOS patients [69], suggesting its role in ovarian function. In fact, increased miR-33b expression inhibited cell growth and enhanced apoptosis by reducing the Wnt-β-catenin signalling pathway [70] and targeting *TGDBRI* and *SMAD7* [69]. Evidence indicates that miR-324 may be linked with PCOS due to its downregulation in PCOS patients compared to controls [71, 72]. Jiang and Ma [72] also found decreased miR-324 expression in their PCOS rat model and discovered that miR-324 may affect apoptosis and GCs proliferation by directly targeting *WNT2B*. Lastly, miR-346 has a poorly understood role in folliculogenesis, although is important in embryogenesis by regulating *EG-VEGF*, crucial for embryo implantation, and by repressing *MMP-2* and *MMP-9* [73]. Previous research in our laboratory indicated that mir-33b, miR-324 and miR-346 were significantly downregulated following THC exposure in sperm [62].

Therefore, this study seeks to investigate the relationship between pharmacologically relevant concentrations of THC and the female reproductive system by assessing DNA methylation and miRNA profiles in GCs following THC exposure. In this investigation, GCs are used as they are an excellent indicator of oocyte health and crucial to oocyte development by providing a suitable microenvironment during oogenesis [74–81]. THC concentrations were chosen based on THC plasma concentrations detected in recreational users and after therapeutic use [82] and are in line with previous research in our laboratory that assessed THC’s effects in gametes and blastocysts [54], and GCs [83]. In addition, THC was detected in follicular fluid of ART patients at a concentration of 0.03243 μM, which is the range of the therapeutic dose (0.032 μM, [THC]) utilized in our study [57].

Herein, we analyzed the effects of THC on epigenetic mechanisms, such as DNA methylation, by measuring 5-methylcytosine (5-mC) and 5-hydroxymethyl cytosine (5-hmC) levels and *DNMT1/3a/3b, TET1/2/3* and *TDG* mRNA and protein expression in GCs. We further looked at the effects of THC on miRNA expression of fertility-associated miRNAs: miR-21, miR-155, miR-346, miR-33b and miR-324. We hypothesized that THC alters epigenetic mechanisms, such as DNA methylation patterns and miRNA profiles, in bovine GCs which might ultimately impact oocyte developmental competency.

## Methods

### Granulosa cells retrieval

Bovine (*Bos Taurus*) ovaries were collected from local abattoirs (Cargill Meat Solutions, Guelph, Ontario, Canada and Highland Packers, Stoney Creek, Ontario, Canada) and transported to the laboratory in sterile warmed saline solution supplemented with penicillin/streptomycin (1%) (University of Guelph, Ontario, Canada) under controlled temperatures of 34–36 °C. GCs used in this study were retrieved and cultured as previously described by Sabry et al. [84]. Briefly, using an aspiration pump set-up, follicles ranging from 2 to 22 mm were aspirated using a sharp 18-gauge needle. GCs were mechanically stripped from aspirated cumulus-oocyte-complexes (COCs) and washed in phosphate-buffered saline (PBS) (Wisent, Saint-Jeane Baptist, QC, Canada) and in 1× Dulbecco’s Modified Eagle Medium (DMEM) (Gibco) containing glutamine (2 mM) (Sigma Aldrich) and penicillin/streptomycin (1%). Cells were resuspended in DMEM supplemented with 20% fetal bovine serum (FBS) (10% total serum-Gibco, 12,483,020) and cultured at 38.5 °C in 5% CO_2_ for 7 days, or until 100% confluent, with media replacement every 48 h. Once confluent, GCs were cryopreserved at passage 1 (P1) in 70% DMEM, 20% FBS and 10% DMSO (Sigma D5879) in liquid nitrogen for further experimentation.

### In vitro granulosa cell culture

Frozen GCs were thawed and resuspended in DMEM supplemented with 20% FBS and incubated at 38.5 °C in 5% CO_2_ for 72 h, or until  >80% confluency was reached. Cells were then trypsinized, resuspended in DMEM supplemented with 10% FBS, split into 6-well plates at a seeding density of 2 × 10^5^ cells, incubated for 24 h, serum starved using OptiMEM™ Reduced Serum Media (Thermo Fisher) for 24 h, and incubated at 38.5 °C for 24 h in one of five treatment groups: a control containing only OptiMEM media, vehicle (1:1:18 ethanol: tween: saline), or GC cell treatment with three clinically relevant concentrations of THC (0.032, 0.32 and 3.2 µM). Cells were snap-frozen in liquid nitrogen and stored at −80 °C for RNA or protein extraction. For all biological replicates, cells were treated at passage 2 (P2), following 120 h of cell culture post-thaw.

### 5-mC and 5-hmC detection by flow cytometry

Following 24-h treatment, GCs were washed, added to DMEM supplemented with 10% FBS and centrifuged at 5000 × *g* for 3 min at 4 °C. Cells were then fixed in 4% PFA (AAA1131336 Fisher Scientific) at 37 °C for 30 min, chilled on ice and centrifuged at 5000 × *g* for 3 min at 4 °C. Cells were resuspended in PBS supplemented with 10% FBS and re-centrifuged under the same conditions. To allow cell permeabilization, cells were resuspended in PBS + 0.1% Triton™ X-100 (Sigma Aldrich) + 5% BSA and gently rocked for one hour at room temperature.

Following cell fixation and permeabilization, 5-mC and 5-hmC levels were detected. A negative isotype control and no stain control were included for both 5-mC and 5-hmC detection. The negative isotype control was included for each primary antibody (5-mC and 5-hmC) to measure the level of fluorescence for non-specific antibody binding. A no stain control was used to control for any background autofluorescence and was used to determine the negative population of cells. Cells were centrifuged at 5000 × *g* for 3 min at 4 °C and resuspended in 50 µl of primary antibody, 5-methylcytocine (5-mC) (Abcam ab10805), (1:100 in PBS + 0.1% Triton™ X-100 + 5% BSA) and were gently rocked for an hour at room temperature. Negative isotype control for 5-mC was added, Mouse igG1, kappa monoclonal isotype control [15-6E10A7] (Abcam ab170190) (1:1000 in PBS + 0.1% Triton™ X-100 + 5% BSA). The no-stain control was incubated in PBS + 0.1% Triton™ X-100 + 5% BSA only. At the end of the 1-h, cells were added to 450 µL of PBS + 0.1% Triton™ X-100 and were centrifuged at 5000 × *g* for 3 min at 4 °C. Cells were then resuspended in PBS + 0.1% Triton™ X-100 and were centrifuged under the same conditions. At this point, cells were resuspended in the secondary antibody, Goat pAb to Ms. IgG (Abcam ab1501133) (1:2000 in PBS + 0.1% Triton™ X-100 + 5% BSA) and placed in the dark at room temperature for 45 min. Cells were then added of PBS + 0.1% Triton™ X-100 and were centrifuged at 5000 × *g* for 3 min at 4 °C. Cells were then resuspended in PBS + 0.1% Triton™ X-100 and were centrifuged under the same conditions. Finally, cells were resuspended in PBS + 5% BSA and were read using Flow Cytometry.

The same protocol was repeated to detect 5-hmC levels using a primary antibody, 5-hydroxymethylcytocine (Abcam AB214728) and a negative isotype control, Rabbit IgG monoclonal [EPR25A] isotype control (ab 172730), both diluted to 1:100 in PBS + 0.1% Triton™ X-100 + 5% BSA. The 5-hmC secondary antibody, Goat pAb to Rb IgG (Abcam ab 150077) was diluted to 1:2000 in PBS + 0.1% Triton™ X-100 + 5% BSA.

To assess 5-mC and 5-hmC levels, flow cytometry was used. Once cells were strained through a 40uM filter (Avantar—VWR) into fluorescence-activated cell sorting (FACS) tubes (Fisher Brand), cells were read using BD Accuri C6 Flow Cytometer and 50,000 events were recorded with a slow fluidics rate. An 8 and 6-peak bead validation was performed before machine use. To visualize 5-mC flow cytometry results, confocal images were taken with Olympus FV1200 Confocal Microscope using DAPI as a counter-stain. Results were analyzed using the Flowjo software on four biological replicates.

### RNA extraction and cDNA synthesis

RNA was extracted from frozen GCs using the RNeasy Plus Micro Kit (Qiagen, Toronto, Canada; 74,034) following manufacturer’s protocol. Briefly, Buffer RLT Plus was added to samples, transferred to gDNA Eliminator Spin Columns and centrifuged. 70% ethanol was added, transferred to RNeasy MinElute Spin columns, and centrifuged. Columns were then washed using RW1 and RPE buffers, and 80% ethanol. Membranes were dried before adding 17 µL of RNAse-free water to elute RNA. RNA concentrations were measured using a Nanodrop 2000c (Thermo Fisher Scientific, Waltham, MA, USA).

mRNA was reverse transcribed (RT) into cDNA using the QuantaBio qScript cDNA Supermix (VWR, Mississauga, Canada; 95,048). Briefly, 4 µL of qScript cDNA Supermix was added to RNA samples. 1000 ng of RNA was then reverse transcribed (RT) in a T100 Thermal Cycler (Bio-Rad, Mississauga, Canada) under the following conditions: 5 min at 25 °C, 30 min at 42 °C, and 5 min at 85 °C. A no template control (NTC) excluding RNA, and no reverse transcription control (NRT) excluding the reverse transcriptase enzyme, were included. cDNA samples were stored at  −20 °C until qPCR analysis.

### Quantitative polymerase chain reaction (qPCR)

The CFX96 Touch Real-Time PCR Detection System (Biorad, 1,725,201) and SsoFast EvaGreen Supermix (Biorad, 1,725,201) were used to determine the mRNA expression of 3 genes involved in DNA methylation *(DNMT1, DNMT3a, DNMT3b)* and 4 genes involved in demethylation *(TET1, TET2, TET3 and TDG)*, following the protocol: 5 min at 95 °C, followed by 44 cycles at 95 °C for 10 s, 60 °C for 10 s and 72 °C for 10 s, as previously described by Saleh et al. [85]. Primer sequences and primer efficiencies are summarized in Table 1. All primers were purchased from Qiagen, except for TDG, which was designed and sequenced [86]. Relative mRNA expression was determined using the efficiency-corrected method (∆∆Ct) with *tyrosine 3-monooxygensae/tryptophan 5-monooxygensae activation protein zeta (YWHAZ)* and *peptidylprolyl isomerase (PPIA)* as reference genes. A calibrator consisting of cDNA from GCs was used to account for inter-run variability. A minimum of five biological replicates were quantified in technical triplicates for each primer set. NTC, NRT and water were measured as RT and qPCR negative controls. mRNA expression profiles were then analyzed using Bio-Rad CFX Maestro software.

**Table 1:** mRNA primer sequences

Gene symbol	Gene full name	GenBank accession #	Product size (bp)	Primer sequence (5’- 3’)	Efficiency (%)	Source
*DNMT1*	DNA (cytosine-5)-methyltransferase 1	NM_182651	136	F: TTAGCACCTCATTTGCCGAGTA R: TAGGTGGAGTCAGGGTTGCTCT	104.2	Sabry et al. [86]
*DNMT3a*	DNA (cytosine-5)-methyltransferase 3a	NM_001206502.1	110	F: GCGTTAGTGACAAGAGGGACA R: AAGGTTCCCCCAGAAGTAGC	100.1	Sabry et al. [86]
*DNMT3b*	DNA (cytosine-5)-methyltransferase 3b	NM_181813.2	103	F: GAAACCAGGACTCGGTCTGA R: GGCCTCGGGTAGAACGTAG	100.4	
*TET1*	Ten-eleven translocation methylcytosine dioxygenase 1	XM_003587999.2	214	F: TTCCCACGGCTCGGTTCT R: RTTTCTGTTCGGAGGCTTTAGTTT	100.9	Sabry et al. [86]
*TET2*	Ten-eleven translocation methylcytosine dioxygenase 2	XM_005207682.1	285	F:AAGGCTGAGGGACGAGAACGA R:GAGACGGAGATGGTATCAAGAATGG	102.0	
*TET3*	Ten-eleven translocation methylcytosine dioxygenase 3	XM_005212473.1	118	F: TCCTTCGGTTGTTCCTGGAG R: TCTTCCGGAGCACTTCTTCC	100.1	
*TDG*	Thymine DNA-glycosylase	NM_001083696.2	159	F: GAACGCGGGCAGCTATTCTC R: GTCTCTCGTGTGGGTTCCTG	99.0	Sabry et al. [86]

### Western blotting

Proteins were extracted from GCs and were lysed in radioimmunoprecipitation assay (RIPA) buffer with protease inhibitors (Bio tool B14001 and B15001) with 4 freeze/thaw cycles using liquid nitrogen, sonication in ice water bath and centrifugation at 12 000 × *g* at 4 °C for 10 min. Protein concentration was determined using Bio-Rad DC Protein Assay Kit (BioRad, Mississauga, ON) following manufacturers’ instructions.

A total of 40 µg of protein was prepared by combining equal volumes of 3× Reducing Buffer plus 5% ß-mercaptoethanol (Sigma Aldrich, M6250). Samples were denatured at 90 °C and loaded onto a 15% polyacrylamide gel in the XCell SureLock Mini-Cell Electrophoresis System (Invitrogen; Burlington, ON, Canada) containing 5X Tris-Glycine Buffer. Gels were run for 2 h at 125 V and transferred onto nitrocellulose membranes (Bio-Rad, 1,620,115) in an Invitrogen wet transfer western blot apparatus (Invitrogen; Burlington, ON, Canada) containing 1X Towbins Buffer, for 2 h at 40 V. Membranes were blocked for 1 h in 5% skim milk in TBST. *DNMT1* primary antibody (1:5000 in 5% skim milk in TBST) was incubated overnight at 4 °C and the anti-rabbit IgG HRP-linked secondary antibody (Cell Signalling Technology, Whitby, ON, Canada; 70,735) (1:2000 in 5% skim milk in TBST) was incubated for 1 h at room temperature. Membranes were then washed, placed in Clarity Western ECL Blotting Substrate (Bio-Rad 170-5060) and imaged using Bio-Rad ChemiDoc XRS + Imaging System. Beta Actin (Cell Signalling Technology, 4967) was used as a loading control (1:2000 in 5% BSA in TBST) and incubated for 1 h at room temperature, followed by incubation with the secondary antibody anti-mouse IgG HRP-Linked antibody (Cell Signalling Technology, 7076) (1:5000 in 5% skim milk in TBST) for 1 h at room temperature. Protein bands were quantified by densitometric analysis using Image Lab software from Bio-Rad and values were expressed as a ratio between *DNMT1* expression and loading control (Beta Actin) for each sample. Four biological replicates were used for this set of experiments.

### MicroRNA analysis

Total RNA was extracted from frozen GCs as previously described. RNA was reverse transcribed using the miRCURY LNA RT Kit (Qiagen 339,340) by first diluting RNA samples to 5 ng/µL in nuclease-free water. Each sample was added to 2 µL of 5× miRCURY SYBR Green RT Reaction Buffer (Qiagen) and 1 µL of 10× miRCURY RT Enzyme Mix (Qiagen). NTC and NRT controls were included. RNA samples were then reverse transcribed under the following conditions: 60 min at 42 °C and 5 min at 4 °C. The resulting cDNA was then stored at −20 °C for qPCR analysis.

miR-21, miR-155, miR-324, miR-346 and miR-33b expressions were quantified by qPCR as described above. Primer sequences are summarized in Table 2. Master Mix composition included 1 µL primer mix, 1 µL RNase-free water and 5 µL 2× miRCURY SYBR Green Master Mix. 3 µL of 1:15 diluted cDNA and 7 µL of Master Mix was added to each well and placed in the CFX96 Touch Real-Time PCR Detection System (Biorad, 1,725,201) under the following conditions: 95 °C for 2 min followed by 40 cycles of 95 °C for 10 s and 56 °C for 60 s, ending with a melt curve analysis from 60–95 °C. Relative microRNA expression was determined using the efficiency-corrected method (∆∆Ct) with miR-93 and miR-132 used as reference genes according to the GeNorm algorithm [87]. A calibrator was used to account for inter-run variability. A minimum of three biological replicates were quantified in technical triplicates for each primer sequence. NTC, NRT and water were measured as RT and qPCR negative controls. microRNA expression profiles were then analyzed using the Bio-Rad CFX Maestro software system.

**Table 2:** microRNA primer sequences

miRNA	Primer ID	Accession #	Primer sequence (5’- 3’)	Efficiency (%)
miR-21	hsa-miR-21-5p	MIMATI0000076	UAGCUUAUCAGACUGAUGUUGA	101.3
miR-155	hsa-miR-155-5p	MIMAT0000646	UUAAUGCUAAUCGUGAUAGGGGUU	99.5
miR-324	hsa-miR-324-5p	MIMAT0000761	CGCAUCCCCUAGGGCAUUGGUG	101.1
miR-346	hsa-miR-346	MIMAT0000826	UGUCUGCCCGCAUGCCUGCCUCU	100.3
miR-93	hsa-miR-93-5p	MIMAT0000093	CAAAGUGCUGUUCGUGCAGGUAG	100.3
miR-132	hsa-miR-132-3p	MIMAT0000426	UAACAGUCUACAGCCAUGGUCG	100.7
miR-33b	hsa-miR-33b-5p	MIMAT0003301	GUGCAUUGCUGUUGCAUUGC	100.3

*miRNA primers were predesigned and validated by Qiagen. Primer efficiencies were tested in this study

### Statistical analysis

GraphPad Prism 6 statistical software was used for all statistical tests. The Shapiro-Wilk Test was used to determine normality within datasets. A One-Way Analysis of Variance (ANOVA) was used on normally distributed datasets and the Kruskal-Wallis test was used on non-normally distributed data. Data were considered statistically significant using a two-tailed p-value < 0.05. A Tukey’s post-hoc test was used on datasets shown to be statistically significant to compare differences among treatment groups. Data were presented as mean ± the standard error of the mean (SEM).

## Results

### 5-mC and 5-hmC Levels in granulosa cells following 24-hour THC exposure

**Fig. 1:**
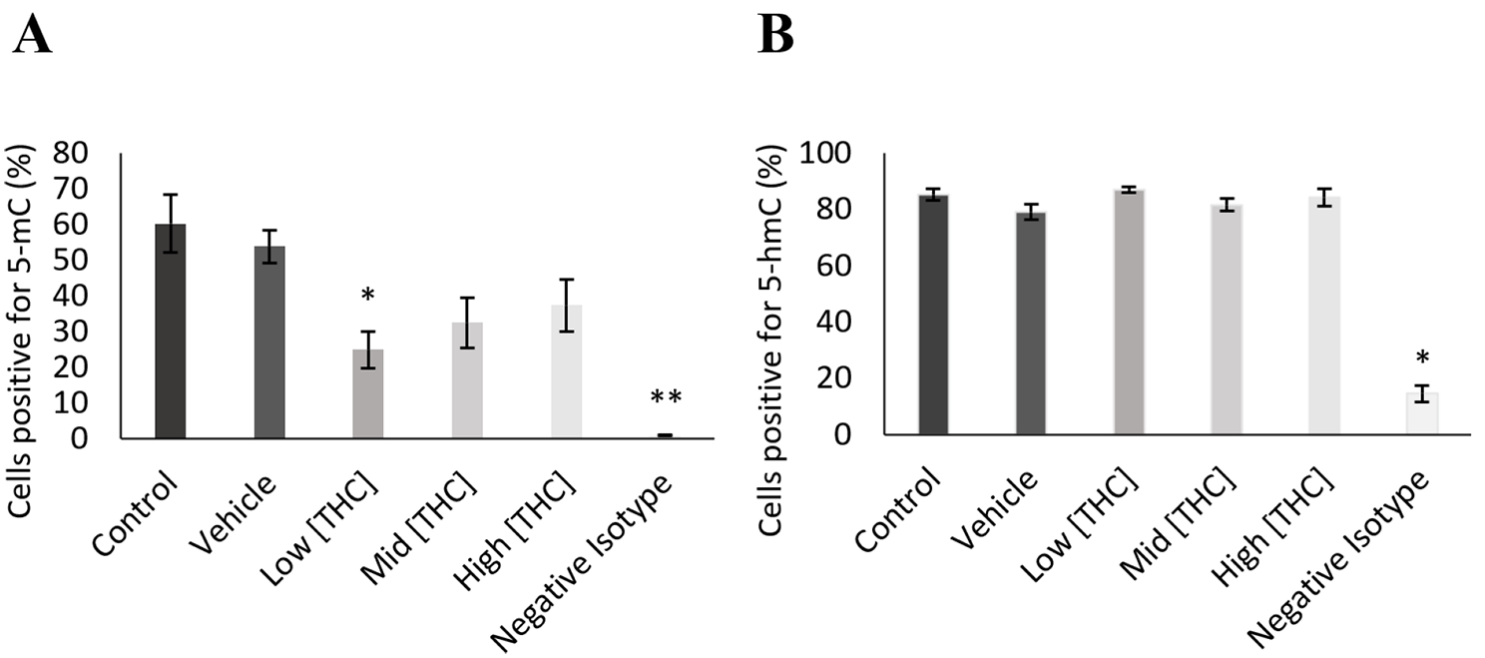
Global methylation levels detected by 5-mC and 5-hmC staining using flow cytometry. **A**) 5-mC (n = 4) and **B**) 5-hmC (n = 4) levels in GCs following 24-hour treatment with low (0.032 µM), mid (0.32 µM), and high (3.2 µM) concentrations of THC. Bars represent ± SEM. *p < 0.05, **p < 0.0005

**Fig. 2:**
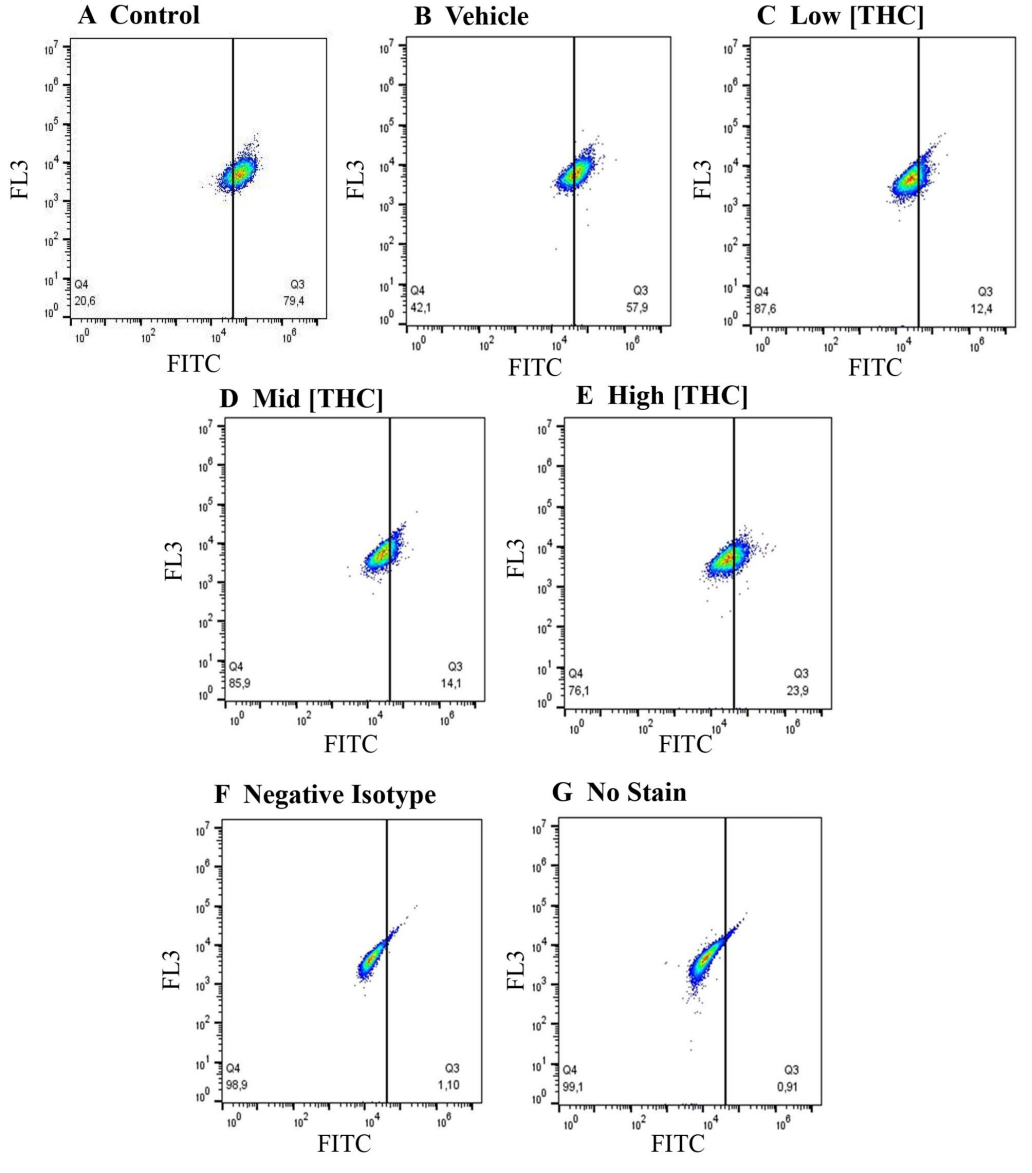
5-mC representative flow cytometry plots. The proportion of no stain (left quadrant) to 5-mC stained (right quadrant) in GCs can be visualized. Plots represent **A**) control, **B**) vehicle, **C**) low THC (0.032 µM), **D**) mid THC (0.32 µM) **E**) high THC (3.2 µM), **F**) negative isotype control and **G**) no stain control

**Fig. 3:**
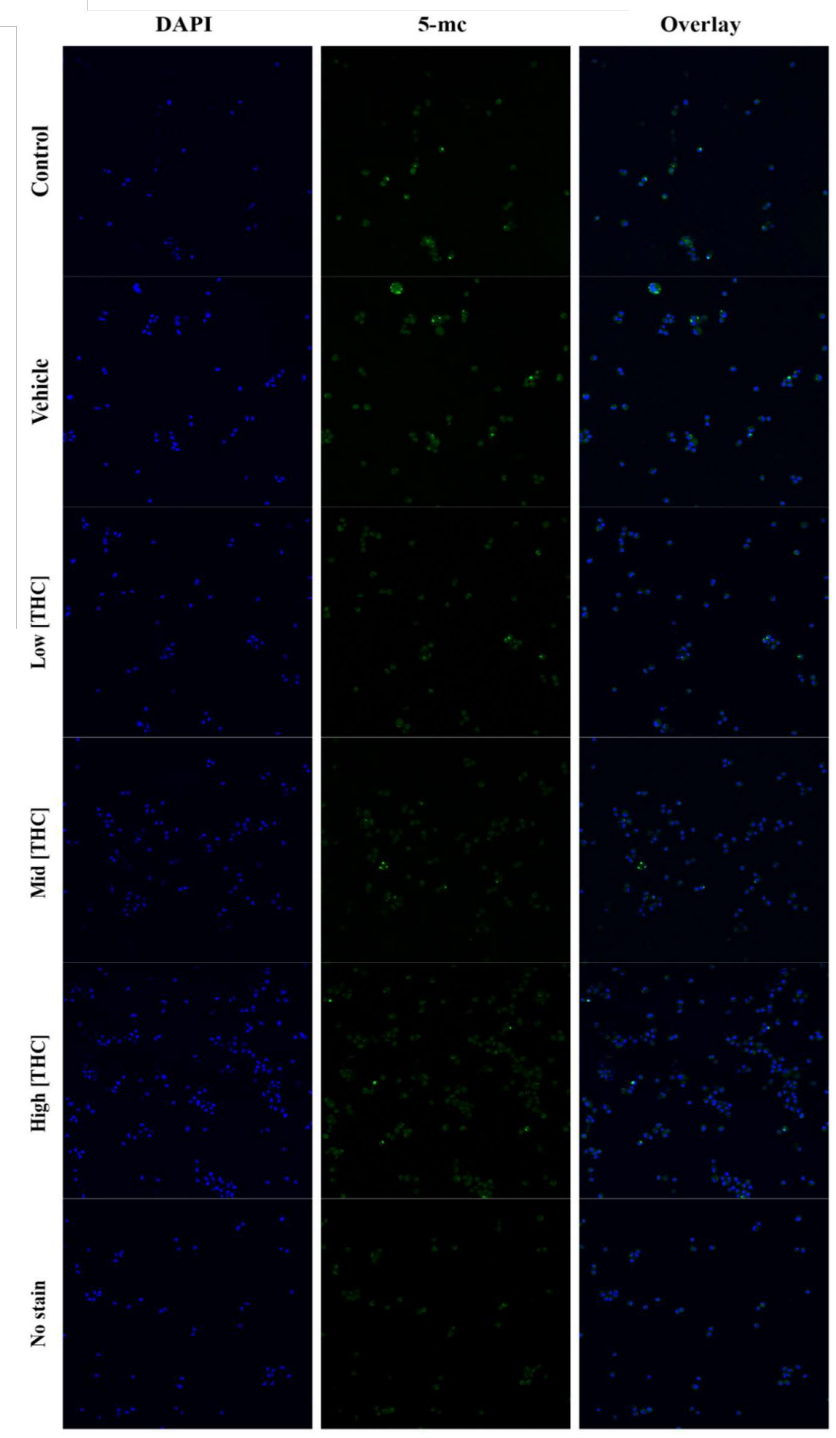
Confocal images of GCs stained with nuclear stain DAPI (blue) and 5-mC antibody (green) following 24-hour treatment with low (0.032 µM), mid (0.32 µM), and high (3.2 µM) concentrations of THC. Images captured using an Olympus FV1200 Confocal Microscope

5-mC and 5-hmC levels were detected in 24 h treated GCs using flow cytometry and gated against no stain control (Figs. 1 and 2). As seen in Fig. 1(A), the 5-mC negative isotype control was significantly reduced compared to control (*p* = 0.0001284, *n* = 4) and 5-mC levels were significantly decreased in the low THC group (*p* = 0.0435, *n* = 4). As depicted in Fig. 1(B), the 5-hmC negative isotype was significantly reduced compared to control (*p* = 0.0154, *n* = 4), however, no differences were observed for 5-hmC levels across treatment groups (Fig. 1B). An overall reduction in 5-mC staining in THC-treated cells can be further visualized in flow cytometry plots (Fig. 2) and confocal images (Fig. 3) taken using Olympus FV1200 Confocal Microscope. As seen in Fig. 2, representative flow cytometry plots were gated into two quadrants- left quadrant representing the negative cell population, and right quadrant showing cells positive for 5-mC. In Fig. 3, blue fluorescence represents nuclear DNA stain (DAPI), while green fluorescence denotes 5-mC staining.

### mRNA and protein expression of DNA methylation genes

**Fig. 4:**
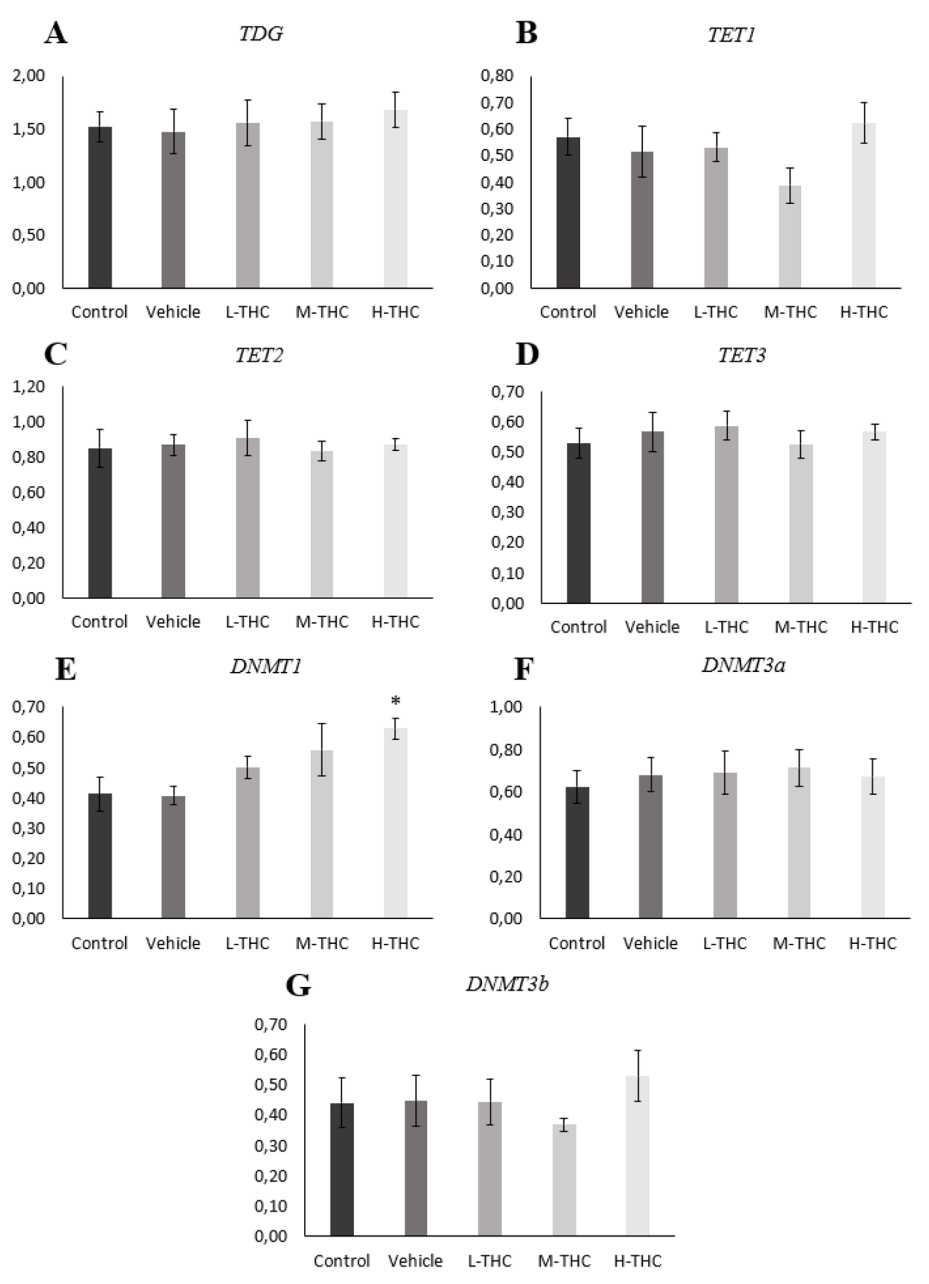
Relative mRNA expression in GCs normalized to housekeeping genes *YWHAZ* and *PPIA*. **A**) *TDG* (n = 6), **B**) *TET1* (n = 6), **C**) *TET2* (n = 6), **D**) *TET3* (n = 6), **E**) *DNMT1* (n = 4), **F**) *DNMT3a* (n = 6) and **G**) *DNMT3b* (n = 5) expression following 24-hour treatment in low (0.032 µM), mid (0.32 µM), and high (3.2 µM) concentrations of THC. Bars represent ± SEM. *p < 0.05

To further evaluate altered 5-mC levels, mRNA expression of key enzymes involved in DNA methylation, including DNMTs *(DNMT1, DNMT3a, DNMT3b),* TET enzymes *(TET1, TET2, TET3)* and *TDG*, was quantified. A minimum of four biological replicates were assessed in GCs following exposure to low (0.032 µM), mid (0.32 µM), and high (3.2 µM) THC. mRNA expression was quantified by qPCR and normalized to reference genes *YWHAZ* and *PPIA*. As seen in Fig. 4(E), *DNMT1* mRNA expression appeared to increase with higher concentrations of THC, however, the increase was statistically significant only following high THC exposure (*p* < 0.05, *n* = 4). In contrast, no significant changes were observed in *DNTM3a, DNMT3b, TET1, TET2, TET3* or *TDG* expression (Fig. 4).

**Fig. 5:**
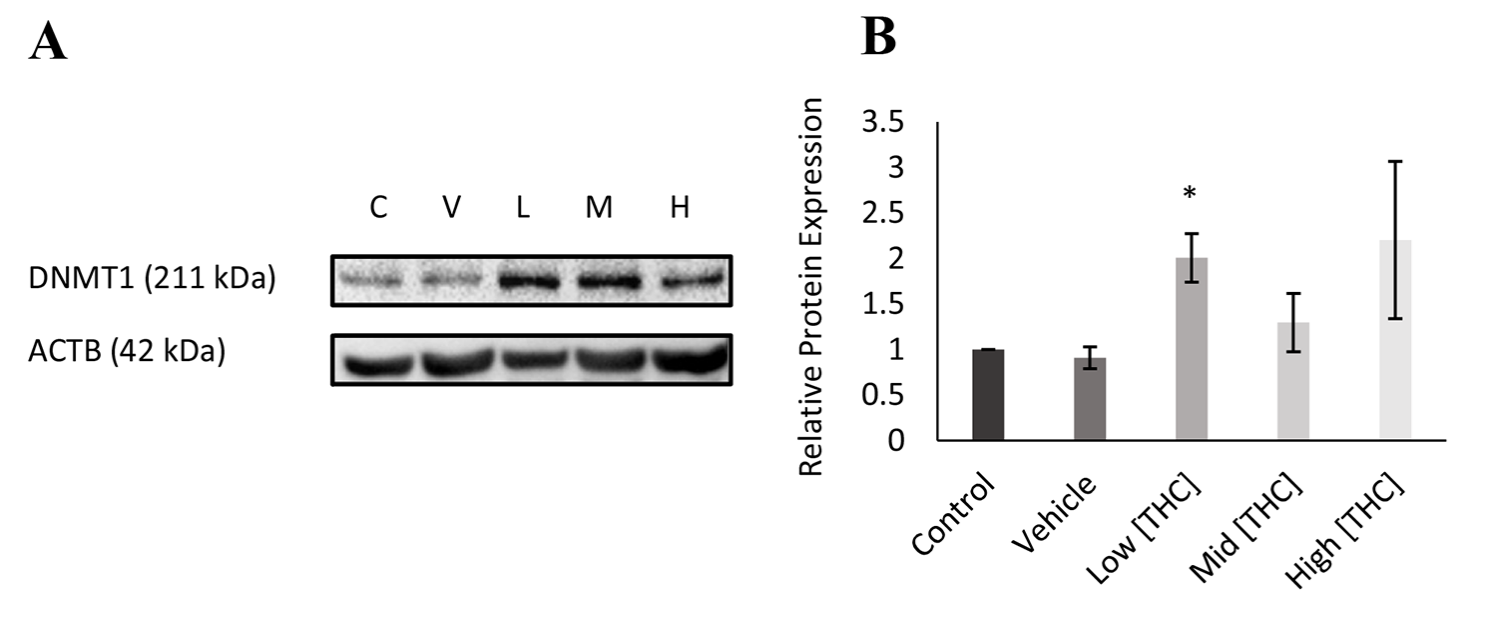
Western blot displaying DNMT1 protein expression relative to loading control ACTB. Expression was qualified in GCs following 24-hour treatment with low (0.032 µM), mid (0.32 µM), and high (3.2 µM) concentrations of THC **A**) DNMT1 protein expression (n = 4) and **B**) densitometry analysis. Bars represent ± SEM. *p = 0.00498. Blots were cropped for representation. Full original uncropped blots can be found in the supplementary material Figure S1

To further analyze the effects of THC on *DNMT1* expression, *DNMT1* proteins were quantified by western blotting with Beta-actin as a loading control. As seen in the densitometry analysis (Fig. 5) and visual representation (Fig. 5), *DNMT1* expression appeared to increase overall following THC exposure, although was only statistically significant following low THC exposure (*p* = 0.00498, *n* = 4).

### MicroRNA expression in THC-treated granulosa cells

**Fig. 6:**
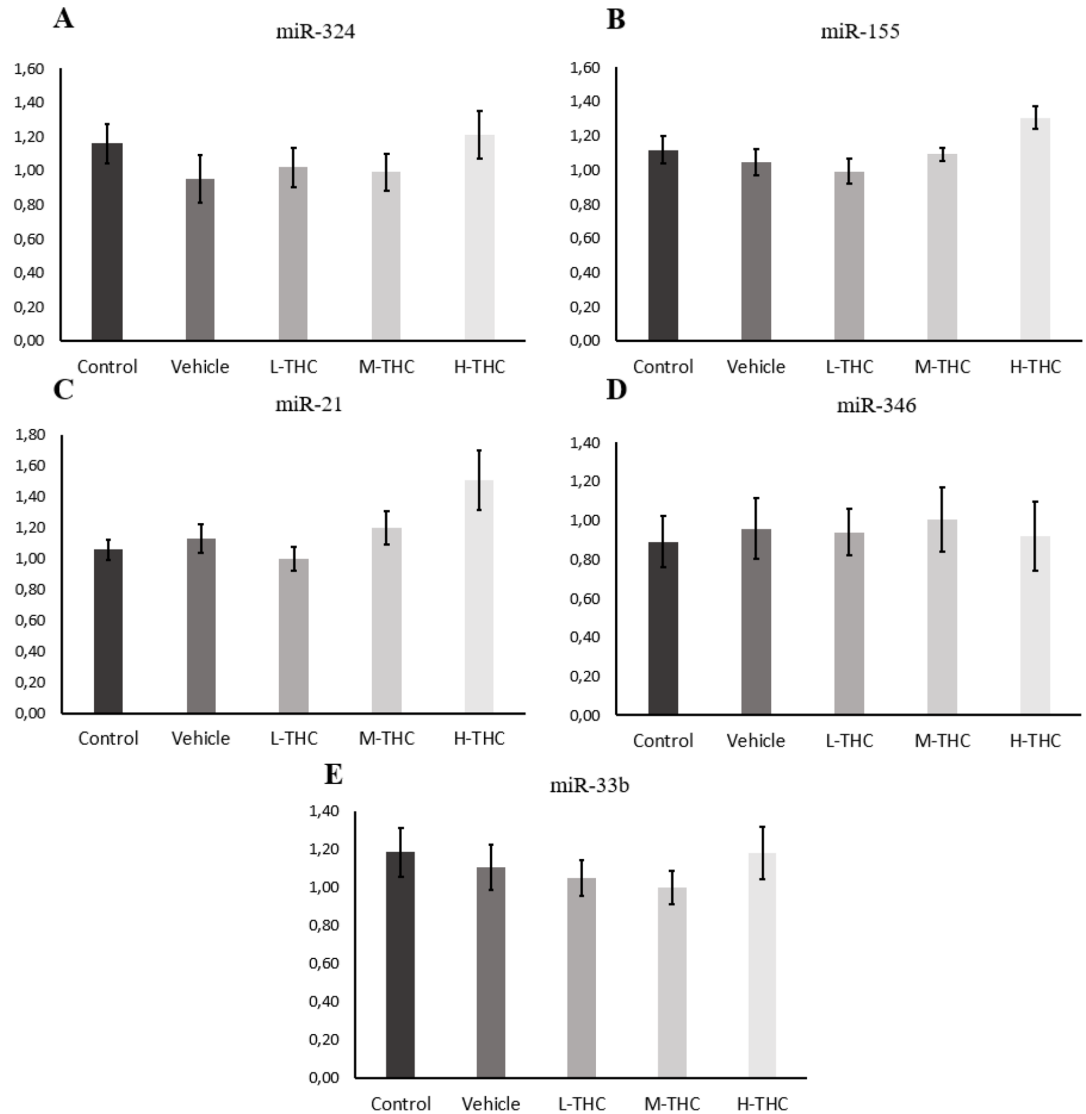
Relative miRNA expression normalized to housekeeping genes miR-93 and miR-132. **A**) miR-324 (n = 6), **B**) miR-155 (n = 3), **C**) miR-21 (n = 6) **D**) miR-346 (n = 6) and **E**) miR-33b (n = 7) expression in GCs following 24-hour treatment with low (0.032 µM), mid (0.32 µM), and high (3.2 µM) concentrations of THC. Bars represent ± SEM

The relative expression of selected fertility associated miRNAs was assessed in GCs following THC exposure. A minimum of three biological replicates was measured by qPCR on the five miRNAs selected (miR-324, miR-155, miR-21, miR-346 and miR-33b) relative to housekeeping miRNAs: miR-93 and miR-132. As seen in Fig. 6(C), miR-21 expression appears to increase with increasing concentrations of THC, although not statistically significant. Overall, no significant changes were seen in miR-324, miR-155, miR-346 and miR-33b expression following THC exposure (Fig. 6).

## Discussion

The goal of this study was to explore the potential effects of THC on epigenetic mechanisms that are known to mediate development, such as DNA methylation and miRNA profiles, in bovine GCs. With the rise in cannabis consumption among reproductive age-women, and the increase in THC potency within cannabis preparation, it is important to further investigate how THC may interact with the ECS within the female reproductive system. The ECS has a prominent role in mammalian reproduction and is present in all female reproductive tissues [15, 33, 40].

To investigate the effects of THC on GCs, three physiologically relevant THC doses were used: a therapeutic low dose (0.032 µM THC), and a recreational mid and high dose, (0.32 µM; 3.2 µM THC, respectively). Therapeutic cannabis is often administered by prescribed drugs such as Marinol ©, which contains a synthetic THC: dronabinol. The low therapeutic level of THC employed in our investigation is reflective of peak plasma concentrations detected 1.5 h after consuming the recommended dose of 10 mg of Marinol © [88]. Likewise, Fuchs Weizman et al. [57] measured THC in follicular fluid of patients undergoing ART in the order of 0.03243 μM, which is comparable with our therapeutic dose of 0.032 μM. Recreational cannabis use is more difficult to predict due to the variability amongst users. As a result, a 10× concentration range of 0.32 and 3.2 µM was employed in this investigation to reflect heavy recreational use. These values were additionally reported by Whan et al. [82] as being representative of blood plasma concentrations found in therapeutic and recreational cannabis users. THC concentrations chosen for this study are also reflective of previously published research from our laboratory investigating THC on sperm parameters [62], GC viability [83], and oocyte and embryo quality [54].

Previously in our laboratory, GCs exposed to therapeutic and recreational doses of THC did not display compromised cell viability or apoptosis [83]. Misner et al. [54] performed a transcriptome analysis which revealed that THC was dysregulating transcript expression in COCs. While it has been documented that THC can cause epigenetic modifications in sperm [11, 58, 62, 89], less is known regarding its epigenetic effects on the female reproductive tract.

Epigenetic mechanisms have an important biological function as they regulate gene expression. The present study focused specifically on DNA methylation and miRNAs. As previously mentioned in more details, DNA methylation is the process of adding methyl groups to cytosine, it is typically mediated by DNMTs and is mainly associated with transcriptional silencing [90, 91]. In addition, DNA also undergoes demethylation, with 5-hmC an intermediate in the demethylation process.

We first assessed global DNA methylation, showing 5-mC levels significantly reduced following low THC exposure. Similarly, a study by Fuchs Weizman et al. [57] found 100 and 500 ng/mL THC-exposed human GCs had decreased DNA methylation by 76.2 and 83.8%, respectively. They further analyzed the effects of a combined treatment of THC, 11-OH-THC and 11-COOH-THC based on follicular fluid concentrations measured in vivo (0.03243, 0.007 and 0.05495 μM, respectively). The combined treatment led to a 37.2% decrease in 5-mC levels following acute (24 h) exposure [57]. In a follow up study, they found THC-exposed GCs had different methylation profiles, with 3679 differentially methylated sites compared to controls [28]. Similar results were also observed by Murphy et al. [11], where 3979 CpG sites were found to be differentially methylated in human sperm exposed to THC, 78% of which had reduced methylation. In contrast, 5-hmC acts as a demethylation intermediate controlled by TET enzymes [92, 93]. No changes in 5-hmC paired with altered 5-mC levels might indicate that THC is affecting DNMTs, but not TET enzymes. To better understand the functional effects of THC-altered 5-mC levels, identifying differentially methylated regions (DMRs) in THC exposed GCs would be valuable and would add significance and strength to this research. We could then isolate which genes are being impacted and how that would affect developmental competency. For instance, by evaluating DMRs in THC exposed rat and human sperm, Murphy et al. [11] identified several KEGG pathways enriched by THC, including Hippo signalling, MAPK signaling, and pathways related to cancer. Hippo signalling is particularly important in follicular activation, growth, maturation and steroidogenesis [94]. In fact, Hippo signalling knockout models led to decreased bovine and mouse GC proliferation and function [95, 96]. Whereas Hippo signaling over-expression resulted in decreased GC proliferation in hens [97, 98], and supported human GC proliferation and growth, but disrupted steroidogenesis [99]. Similarly, MAPK activity in cumulus GCs plays an important role in meiotic resumption, ovulation and luteinization [100, 101]. DMRs were also identified by Fuchs Weizman et al. [28] in THC-exposed GCs. These regions were associated with epigenetic modifications, transcription factors, cell proliferation, apoptosis, post-translational modifications, and extra cellular matrix remodeling [28]. Of the DMRs discovered, they found 47% were hypermethylated, while 53% were hypomethylated [28]. The reported total 5-mC levels in this investigation rather than DMRs, do not account for the percentage of DNA being hypermethylated versus hypomethylated.

Previous work in our laboratory by Misner et al. [54] observed decreased connexin 37 and 43 levels in COCs exposed to low THC, while no changes were detected following mid or high THC exposure. In addition, they found 62 differentially expressed genes in the low THC group compared to controls, whereas only a handful of genes were differentially expressed in the mid and high groups [54]. Gene expression is related to the accessibility of transcription machinery to DNA, partially controlled by methylation. Thus, paired with the results in the present study, upregulated gene expression may be a result of decreased 5-mC levels. If transcript levels were increased in COCs exposed to THC, perhaps there is an increase in transcripts being sent from the surrounding GCs due to decreased DNA methylation. The bidirectional communication between the oocyte and its surrounding GCs is essential for folliculogenesis, oocyte maturation and competence acquisition [79, 81, 102]. Throughout folliculogenesis, the oocyte and surrounding GCs transfer molecules, including ions, cAMP, metabolites, amino acids and RNA transcripts [81].

Global DNA methylation is governed by establishing new methylation patterns, maintaining methylation patterns during cell replication and active demethylation. Together, DNMTs and TETs/TDG establish, maintain and erase CpG methyl marks, contributing to overall gene expression [90]. The detected increase in *DNMT1* mRNA and protein expression may be indicative of increased cell replication, but previous research in our lab found no significant changes in cell counts following THC exposure [83]. Cells might be increasing *DNMT1* transcripts to compensate for reduced 5-mC marks following THC exposure. Previous research by Vassall et al. [101] found a significant increase in *DNMT1* in the medaka fish ovary following THC treatment. Although our results showed no changes in *DNMT3a* or *DNMT3b* expression, Vassall et al. [103] found decreased *DNMT3a*, while Fuchs Weizman et al. [57] found reduced *DNMT3b* expression, both in vivo and confirmed in vitro, in THC exposed human GCs. Smith et al. [104] also found *TET3* upregulated in blood lymphocytes paired with detected THC levels, following acute cannabis smoking. These varied results are likely from different study designs, such as exposure route (in vitro vs inhalation), time of exposure, different models and the effects of other cannabis components, such as CBD. Therefore, future studies looking at CBD effects and a combination of THC and CBD effects would be needed to overcome the limitation of assessing only the role of THC.

In addition to DNA methylation, *DNMT1* is directly involved in DNA damage repair to maintain chromosome integrity [105–107]. Following the recruitment of *DNMT1* to DNA repair sites, *DNMT1* regulates the rate of ATR signaling [107]. ATR is a major regulator of the DNA damage response in cells during replication as it controls replication origin firing, replication fork stability, cell cycle checkpoints, and DNA repair [108]. Therefore, it is plausible that THC might be affecting DNA integrity, resulting in increased *DNMT1* expression.

THC acts on the ECS, which in turn activates multiple pathways including P13K/AKT, MAPK and cAMP [27, 32]. Notably, the AKT1 pathway stabilises *DNMT1* expression, and a rise in *DNMT1* expression depends on *AKT1* [109, 110]. Likewise, P1K3/AKT signaling can increase *DNMT1* expression [111], which can then lead to decreased E-cadherins [110]. E-cadherins promote cell-to-cell contact with the surrounding GCs [112], which maintains follicular integrity [113]. It can be speculated that THC may be altering the P1K3/AKT pathway, subsequently resulting in increased *DNMT1* expression.

To further elucidate the epigenetic effects of THC on GCs, we assessed fertility-associated miRNAs, important in cell survival, apoptosis, cell growth and proliferation during folliculogenesis. THC may be increasing miR-21 expression in a dose dependent manner, although these changes were not statistically significant. No significant changes were observed in the other miRNAs analyzed, contradicting previous work in our laboratory on THC-treated sperm showing a decrease in miR-33b, miR-324, and miR-346 expression [62], suggesting a different impact of THC on the female and male gametes. To our knowledge, no other studies have documented the effects of THC on these miRNAs in gametes, making this research novel and even more valuable.

Similar to our observations, Jackson et al. [114] and Sido et al. [115] showed that both AEA and THC significantly upregulated miR-21 in mouse lymphocytes, suggesting that miR-21 might likely be a target of the ECS. Our results indicated no significant changes in miR-155 expression, but other studies have shown a decrease in miR-155 expression from combined exposure to THC and CBD [116], and to CBD alone [117]. Interestingly, Juknat et al. [117] found that CBD had a greater effect on miRNA profiles compared to THC. Paired with our findings, this suggests THC has a smaller effect on miRNA expression compared to other epigenetic mechanisms, such as DNA methylation. It would be worth investigating additional miRNAs important in folliculogenesis, including miR-212, miR-214, miR-99a, miR-100 and miR-218. miR-212 and miR-214 are synthesized by GCs and sent to the oocyte for meiotic resumption, while miR-99a, miR-100 and miR-218 are involved in follicular maturation [81, 118]. This would allow to overcome one of the limitations of this study, that is the restricted number of target miRs investigated.

Epigenetic mechanisms often work together to regulate gene expression, therefore THC’s effects should be assessed on other epigenetic modulators, such as histone modifications. The interaction of histones and DNA affects chromatin integrity, which in turn regulates gene expression [119]. Histone modifications are important in oocyte development [120] and can be altered from THC exposure [119, 121–123]. In fact, Fuchs Weizman et al. [28] found DMRs from THC exposure were associated with histone methyltransferase SMYD3 and ZFP37, which alter histone acetylation and methylation. Another possible avenue for this research might consist in investigating THC effects on other types of ncRNAs, such as long-noncoding RNAs (lncRNAs), short interfering RNAs (siRNAs) and circular RNAs (circRNAs), as these ncRNAs have been proven to be involved in primary ovarian insufficiencies [124], in post transcriptional regulation during oocyte maturation [125], in meiotic resumption, spindle formation and chromosome alignment [126] and play a role in epigenetic regulation, being correlated to oocyte and embryo competency [127, 128].

As the oocyte and its surrounding GCs communicate in a bidirectional manner, it is important to address how THC’s effects on DNA methylation in GCs impact the oocyte. The epigenome, including DNA methylation, is passed down to future progeny. Hofmeister et al. [125] discovered that 99.998% of the methylated genome was effectively inherited across multiple generations, concluding that DNA methylation is extremely stable across generations, thus, THC’s ability to alter DNA methylation can produce transgenerational impacts.

The potential consequences of perturbations in DNA methylation could be detrimental to oocyte growth and development. Changes in DNA methylation patterns could lead to enriched pathways such as hippo signalling and MAPK signalling [91]. These pathways are important in follicular activation, growth [92, 93], meiotic resumption, ovulation and luteinization [97, 98]. Any disruptions to these critical processes may lead to cellular apoptosis, interrupt steroidogenesis [96] and disrupt follicular integrity [108]. Therefore, oocyte growth and maturation may be negatively compromised by such epigenetic changes.

The strength of this research lays on the clinical relevance of the doses used and the novelty in the investigation of specific microRNAs and epigenetics changes that can have an effect not only on the individual, but also on generations to come. As previously mentioned the oocyte and the surrounding granulosa cells are constantly in a bidirectional communication, therefore further studies looking at these epigenetic changes directly on the oocytes will add significance to this research that has the limitation of being conducted only in granulosa cells. However, this study is still extremely important because of the lack of knowledge and studies in the field.

## Conclusions

In summary, the results presented in this paper support the hypothesis that THC alters epigenetic mechanisms, such as DNA methylation patterns, but does not affect miRNAs in bovine GCs. Altered DNA methylation will subsequently lead to changes in gene expression, which may affect developmental processes that require precisely regulated gene activity, ultimately impairing oocyte competence. Overall, the importance of this research lays in advancing our understanding of how cannabis use may affect fertility, ultimately providing scientific evidence to better advise patients undergoing ARTs about the effect(s) of cannabis consumption. Furthermore, this information will be valuable in developing guidelines similar to the ones established for other recreationally-used substances, such as alcohol and tobacco.

### Electronic supplementary material

Below is the link to the electronic supplementary material.

Supplementary Material 1

Supplementary Material 2

## Data Availability

The datasets used and/or analysed during the current study are available from the corresponding author on reasonable request.

## Abbreviations

AEA: Anandamide
ANOVA: Analysis of variance
ART: Assisted reproductive technology
BSA: Bovine serum albumin
CB1: Endocannabinoid receptor 1
CB2: Endocannabinoid receptor 2
CBD: Cannabidiol
cDNA: Complementary DNA
circRNA: circular RNAs
COC: Cumulus-oocyte-complex
DMEM: Dubecco’s modified Eagle’s medium
DMRs: Differentially methylated regions
DNMT: DNA methyltransferase
eCB: Endocannabinoids
ECS: Endocannabinoid system
ECL: Enhanced chemiluminescence
FAAH: Fatty acid amine hydrolase
FBS: Fetal bovine serum
GC: Granulosa cell
IVF: In vitro fertilization
lncRNA: Long non-coding RNAs
miRNA: Micro-ribonucleic acid
mRNA: Messenger ribonucleic acid
PBS: Phosphate-buffered saline
PCOS: Polycystic ovary syndrome
PFA: Paraformaldehyde
qPCR: Quantitative polymerase chain reaction
RIPA: Radioimmunoprecipitation assay
RT: Reverse transcription
SEM: Standard error of the mean
siRNA: Small interfering RNAs
sncRNA: Small non-coding RNAs
TBST: Tris buffered saline with tween
TDG: Thymine DNA glycosylase
TET: Ten-eleven translocation
THC: Delta-9-tetrahydrocannabinol
2-AG: 2-arachidonyl glycerol
5-mC: 5-methyl cytosine
5-hmC: 5-hydroxymethyl cytosine
