# Supplementary material for: DNA methylation, but not microRNA expression, is affected by in vitro THC exposure in bovine granulosa cells
Source: BMC Pharmacol Toxicol. 2024 Jul 15;25:42. doi: 10.1186/s40360-024-00763-5 (PMC11247865; doi:10.1186/s40360-024-00763-5)
Supplement: Supplementary file 1 — Supplementary Material 1 [file 40360_2024_763_MOESM1_ESM.docx]

**Supplementary Materials:**

**Reference Gene Selection**

Appropriate reference genes were selected following THC treatment using the reference gene software, geNorm and qPCR. The five candidate genes considered were the following: *Beta-2 microglobulin* *(B2M),* *Glyceraldehyde 3-phosphate dehydrogenase* *(GAPDH),* *Beta-Actin* *(ACTB),* *Tyrosine 3-monooxygenase/tryptophan 5-monooxygenase activation protein zeta* *(YWHAZ)* and *Peptidylprolyl isomerase A* *(PPIA)* (Supplementary Table 1). *YWHAZ* and *PPIA* were selected as the most stable (Supplementary Figure 1).

Supplementary Table 1. mRNA housekeeping primer candidates

| **Gene Symbol** | **Gene Full Name** | **GenBank Accession #** | **Primer Sequence (5’-3’)** | **Efficiency (%)** | **Source** |
| --- | --- | --- | --- | --- | --- |
| *GAPDH* | Glyceraldehyde 3-phosphate dehydrogenase | NM_001034034.2 | F:TTCCTGGTACGACAATGAATTTG  R: GGAGATGGGGCAGGACTC | 99.50 | Ferris et al., 2016 |
| *ACTB* | Beta-actin | NM_173979.3 | F: CCTTCCTGGGCATGGAATCCT  R:TCTTCATTGTGCTGGGTGCC | 97.00 | Tscherner, 2017 |
| *YWHAZ* | Tyrosine 3-monooxygenase/ tryptophan 5-monooxygenase activation protein zeta | NM_174813.2 | F: GCARCCCACAGACTATTTCC  R: GCAAAGACAATGACAGACCA | 99.0 | Sharma, 2016 |
| *PPIA* | Peptidylprolyl isomerase A | NM_178320.2 | F: TCTTGTCCATGGCAAATGCTG  R: TTTCACCTTGCCAAAGTACCAC | 102.3 | Ferris et al., 2016 |
| *B2M* | Beta-2 microglobulin | NM_004048.2 | F: ACGCTGAGTTCACTCCCAACAGCAA  R: TCGATGGTGCTGCTTACAGGTCTCG | 100.0 | Baddela et al., (2014) |


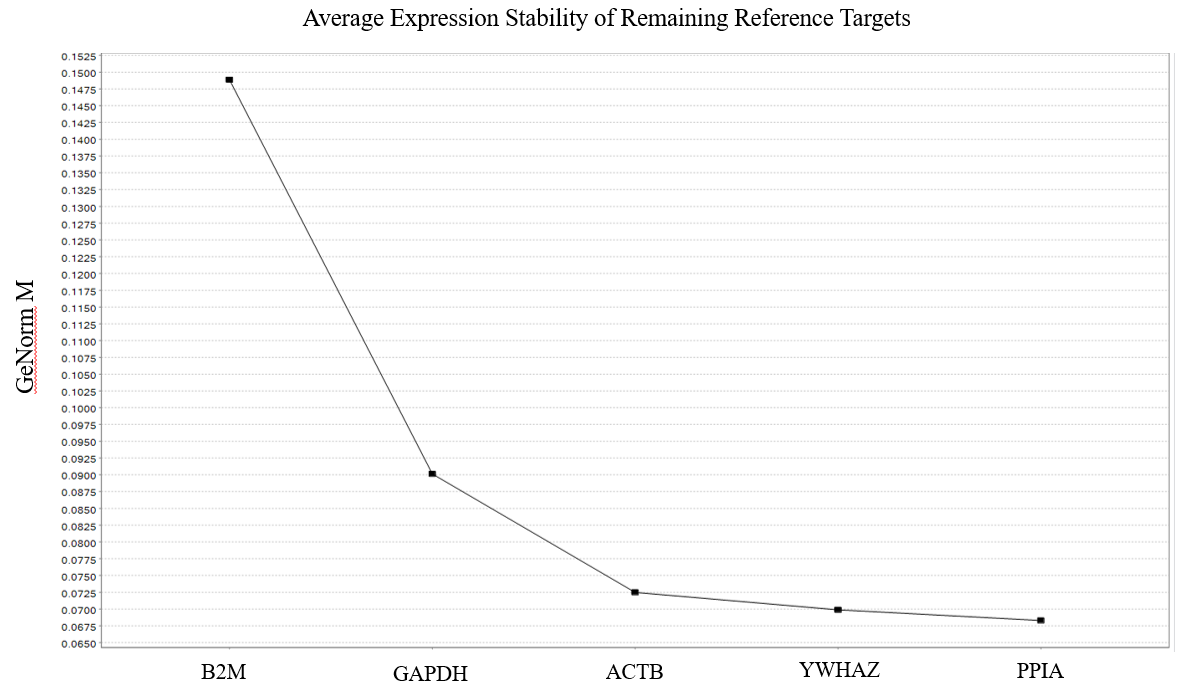


**Supplementary Figure 1 -** mRNA reference gene selection for THC-treated GCs using GeNorm (n=3). Reference gene candidates include *B2M, GAPDH, ACTB, YWHAZ* and *PPIA*. All candidate genes were below the cut-off value for stability (M=0.5), although *YWHAZ* and *PPIA* (M<0.07) were most stable.

**Primer Efficiency Testing**

Primer efficiencies were tested for each primer set via standard curves using qPCR. Standard curves were established using the following serial dilution: 50 ng, 25ng, 12.5 ng, 6.25ng, 3.125ng, 1.56ng, 0.78ng, 0.39ng. The CFX96 Touch Real-Time PCR Detection System (Biorad, 1725201) and SsoFast EvaGreen Supermix (Biorad, 1725201) were used following the protocol: 5 minutes at 95°C, followed by 44 cycles at 95°C for 10 seconds, 60°C for 10 seconds and 72°C for 10 seconds. miRNA primer efficiencies were tested following the same serial dilution as mentioned above, under the following conditions: 95℃ for 2 minutes followed by 40 cycles of 95℃ for 10 seconds and 56℃ for 60 seconds. Only efficiency values between 90-100% were accepted. Primer efficiencies are summurized in Table 1, Table 2 and supplementary Table 1.


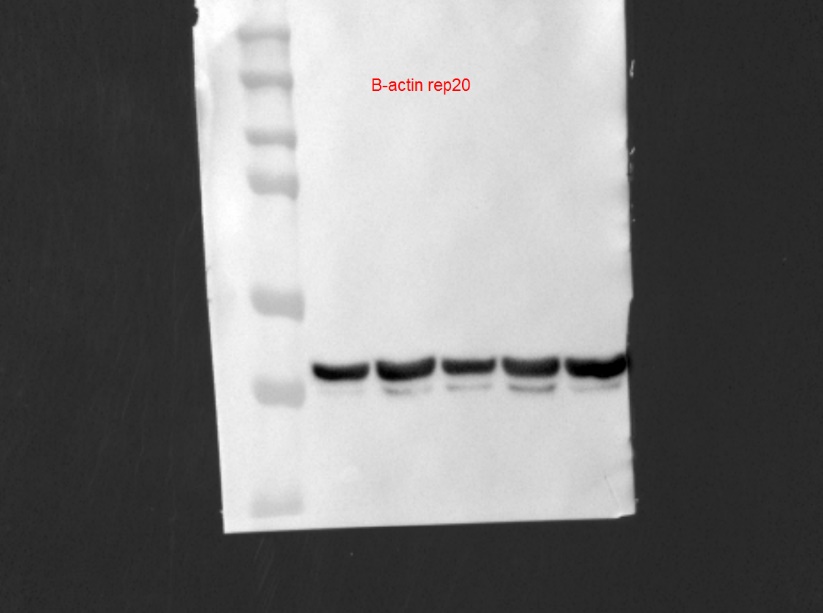


42 kDa

Mid

[THC]

High

[THC]

Low

[THC]

Vehicle

Control


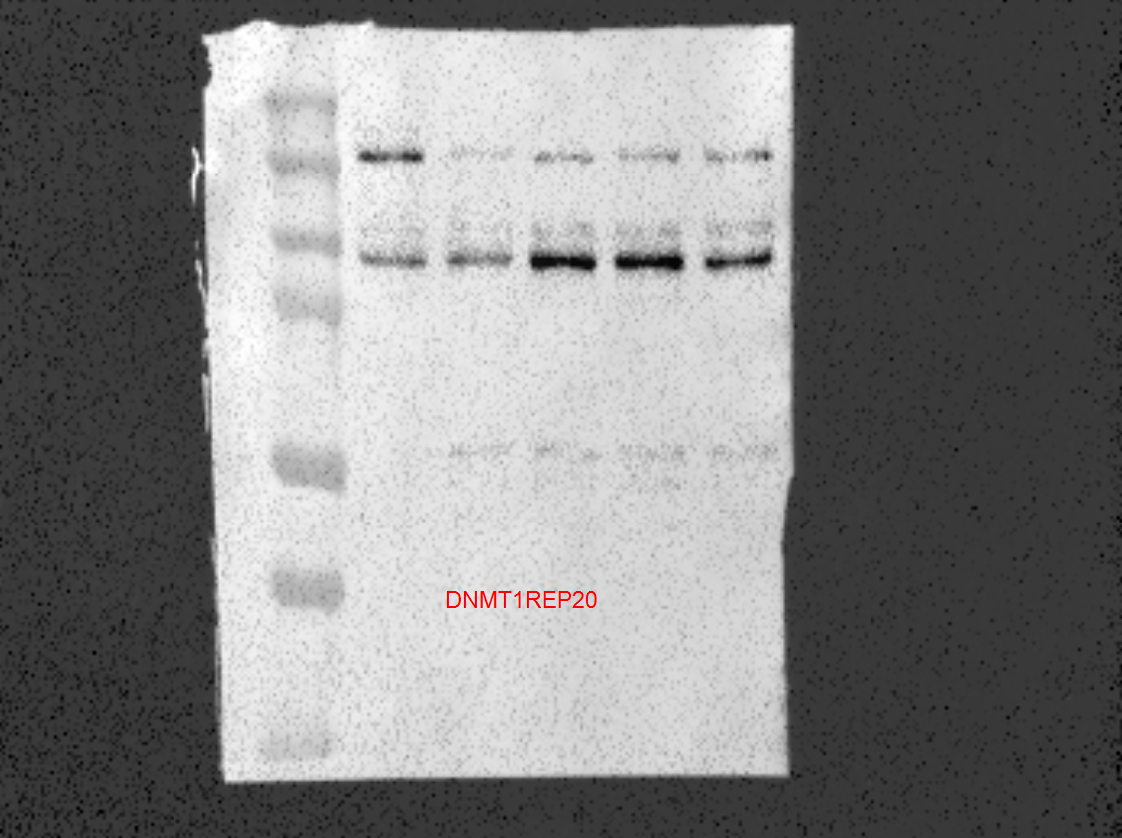


211 kDa

Control

Low

[THC]

Mid

[THC]

High

[THC]

Vehicle

**Supplementary Figure 2 – Original Western Blots.** The original uncropped western blots as represented in the manuscript in Figure 5.
