# Supplementary material for: DNA methylation, but not microRNA expression, is affected by in vitro THC exposure in bovine granulosa cells
Source: BMC Pharmacol Toxicol. 2024 Jul 15;25:42. doi: 10.1186/s40360-024-00763-5 (PMC11247865; doi:10.1186/s40360-024-00763-5)
Supplement: Supplementary file 2 — Supplementary Material 2 [file 40360_2024_763_MOESM2_ESM.docx]

**Western Blots Used for Analysis**

1. **DNMT1**


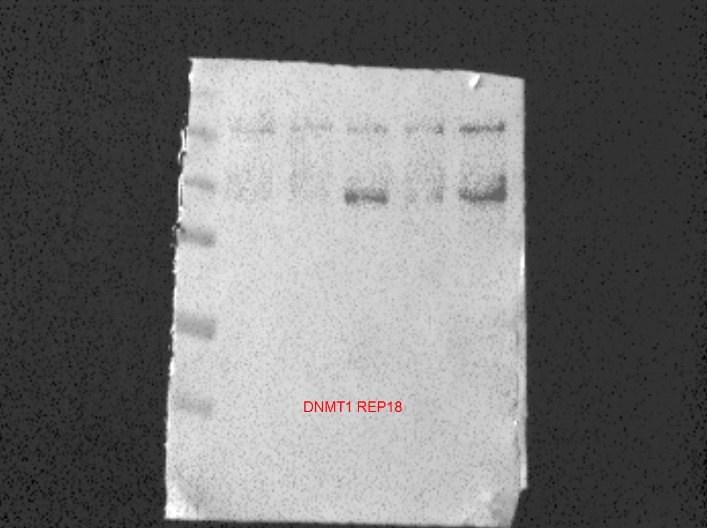

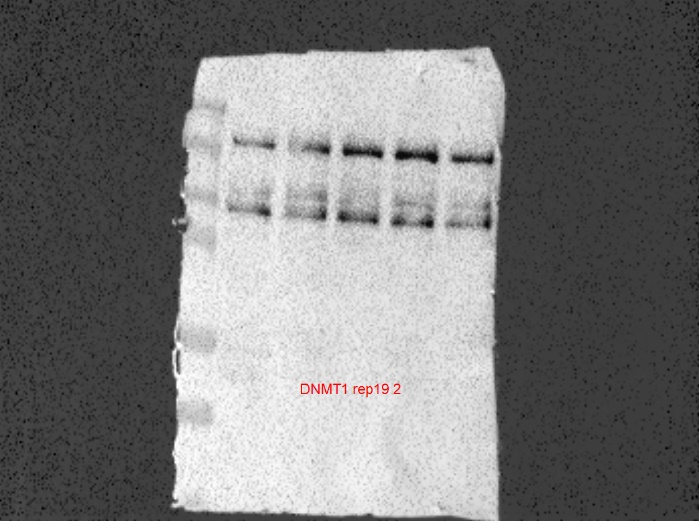

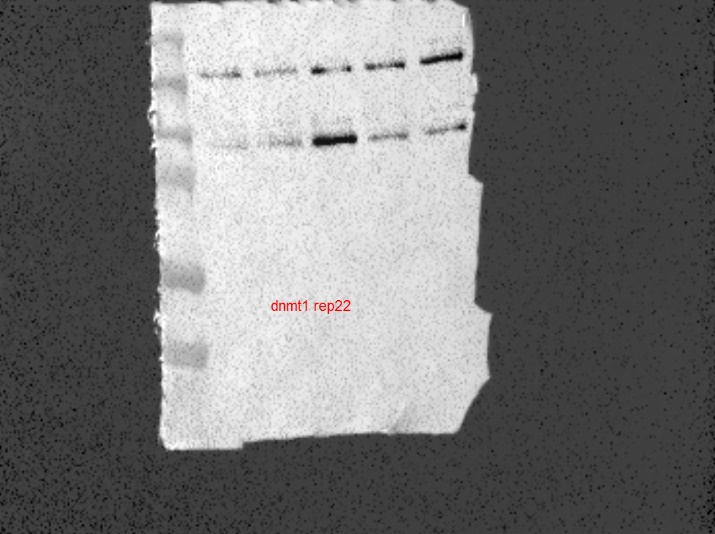

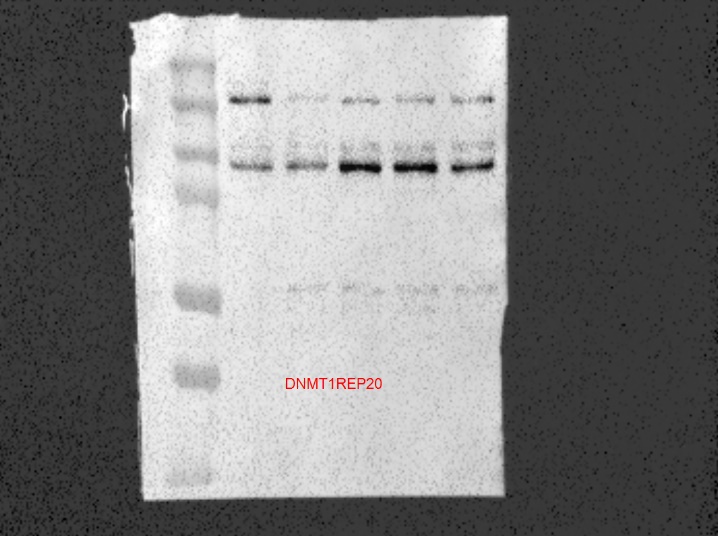


211 kDa

211 kDa

211 kDa

Cont. Veh. Low Mid High

Cont. Veh. Low Mid High

Cont. Veh. Low Mid High

Cont. Veh. Low Mid High

211 kDa

1. **B-actin**


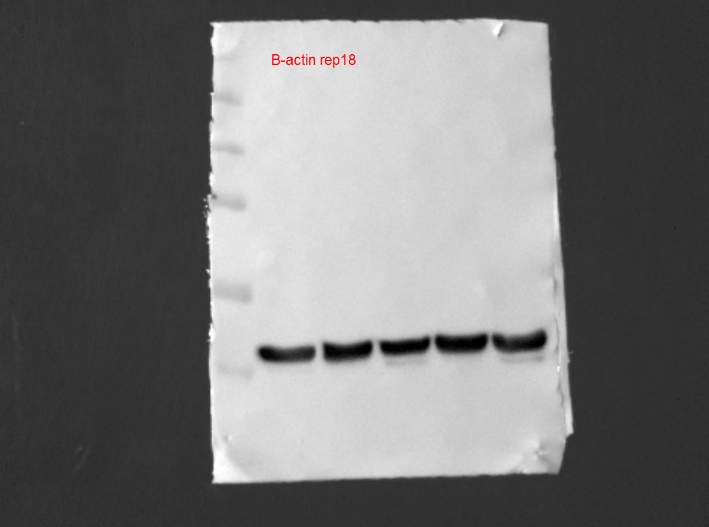

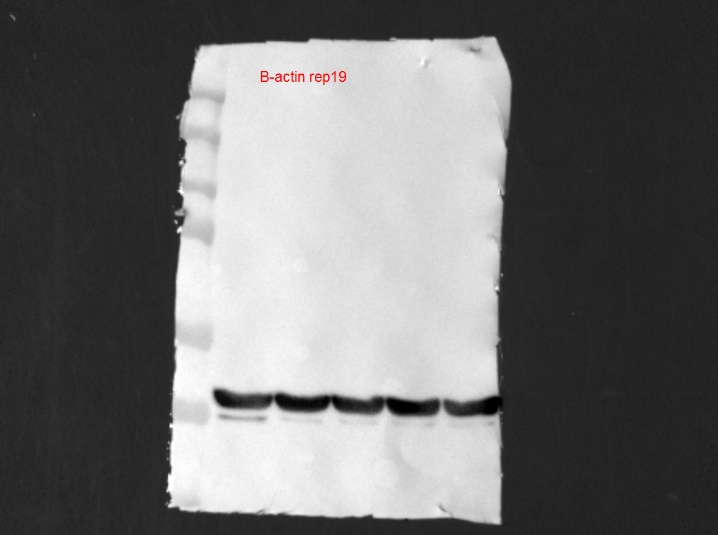


Cont. Veh. Low Mid High

Cont. Veh. Low Mid High

42 kDa

42 kDa


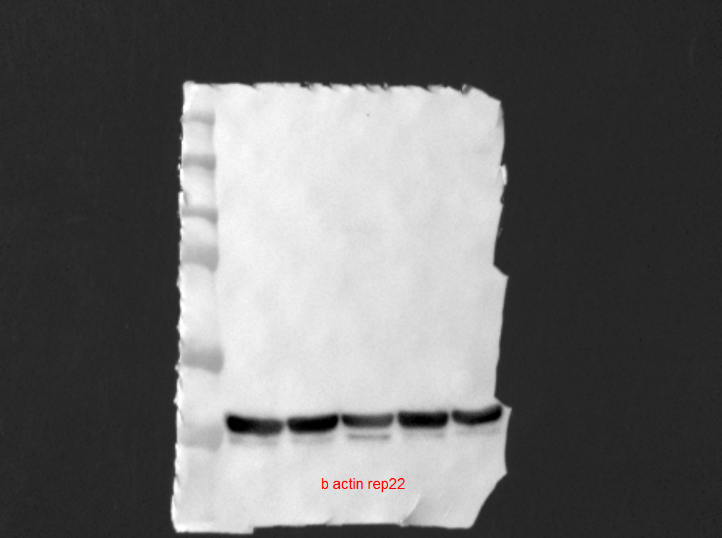

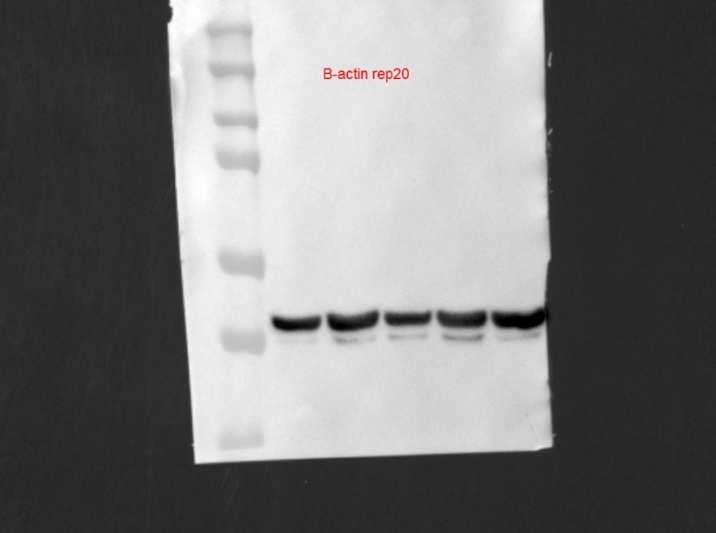


Cont. Veh. Low Mid High

Cont. Veh. Low Mid High

42 kDa

42 kDa
